# Expansion of a retrovirus lineage in the koala genome

**DOI:** 10.1073/pnas.2201844119

**Published:** 2022-06-13

**Authors:** Mette Lillie, Jason Hill, Mats E. Pettersson, Patric Jern

**Affiliations:** ^a^Science for Life Laboratory, Department of Medical Biochemistry and Microbiology, Uppsala University, SE-751 23 Uppsala, Sweden

**Keywords:** endogenous retrovirus, koala, polymorphism, evolution

## Abstract

Retroviruses have left their legacy in host genomes over millions of years as endogenous retroviruses (ERVs), and their structure, diversity, and prevalence provide insights into the historical dynamics of retrovirus–host interactions. In bioinformatic analyses of koala (*Phascolarctos cinereus*) whole-genome sequences, we identify a recently expanded ERV lineage (*phaCin-β*) that is related to the New World squirrel monkey retrovirus. This ERV expansion shares many parallels with the ongoing koala retrovirus (KoRV) invasion of the koala genome, including highly similar and mostly intact sequences, and polymorphic ERV loci in the sampled koala population. The recent *phaCin-β* ERV colonization of the koala genome appears to predate the current KoRV invasion, but polymorphic ERVs and divergence comparisons between these two lineages predict a currently uncharacterized, possibly still extant, *phaCin-β* retrovirus. The genomics approach to ERV-guided discovery of novel retroviruses in host species provides a strong incentive to search for *phaCin-β* retroviruses in the Australasian fauna.

## Significance

The endogenous retrovirus (ERV) record in host DNA left by retrovirus infections in the germline over millions of years allows studies of historic retrovirus–host dynamics, which is increasingly relevant considering contemporary zoonotic viral transmissions and mounting efforts in conservation management of threatened species. Applying bioinformatics on whole-genome sequencing and host population data allow us to draw parallels between ERV lineages, such as the *phaCin-β* ERVs, related to the squirrel monkey retrovirus that we identified in koala, and the currently invading koala retrovirus. Based on unfixed ERVs in the koala population and divergence comparisons between these two ERV lineages, it is conceivable that active *phaCin-β* retroviruses remain to be discovered in the Australasian fauna.

Retroviruses are a diverse group of RNA viruses that convert their genomes to proviral DNA and integrate permanently into the host nuclear DNA, in order to produce virus progeny. Sporadic integrations in the host germline over millions of years have left a record of historical retrovirus activity as inherited endogenous retroviruses (ERVs) in contemporary host genomes (1, 2). ERVs are subject to the molecular evolution of the host genome, and are frequently inactivated by mutation or recombination between the proviral long-terminal repeats (LTRs), causing loss of proviral genes and leaving a solitary LTR at the integration site. The functional consequences of ERVs are diverse, depending on the genomic architecture of the integration site and modifications to the proviral sequence (1, 2). The completeness of ERV structure, diversity, and prevalence throughout the host genome can provide valuable insights into the historical dynamics of retrovirus–host interactions.

Here, we identify a recently expanded koala (*Phascolarctos cinereus*) ERV lineage, *phaCin-β*, related to the New World squirrel monkey retrovirus (SMRV). SMRV can replicate in dog, mouse, and human cells, but hybridization studies were unable to detect SMRV in other New World monkeys, Old World monkeys, apes, or humans. Although more recent genomic screening identified SMRV-related ERVs in other New World hosts (3), this lineage’s history remains obscure. Parallels can be drawn between the *phaCin-β* expansion and the ongoing koala retrovirus (KoRV) invasion, whereby many highly similar ERV loci with intact structures can be identified in the koala genomes. KoRV has attracted considerable research interest and infections have been linked to wide-ranging disease outcomes, including cancers and opportunistic infections. KoRV prevalence varies along the koala distribution, ranging from near 100% prevalence in the north to low prevalence in southern island populations (4–6). Among the KoRV subtypes (A–K) (7, 8), only KoRV-A is known to successfully colonize the koala germline as endogenous enKoRV (7, 9–12). In contrast to typical ERVs, which date back millions of years (13), enKoRV appears younger than 50,000 y (10, 14). The similar expansion of *phaCin-β* ERVs presented in this study indicates that the koala has been subject to at least two distinct retroviral invasions across its population in recent history. These findings warrant a search for novel *phaCin-β* retroviruses in Australasian fauna and emphasize koala as a model for investigating susceptibility to retrovirus infection and germline invasion. Our results also highlight the value of studying ERV diversity and strength of ERV-guided search for recent, even ongoing, retroviral invasion of host populations.

## Results

**Fig. 1.**
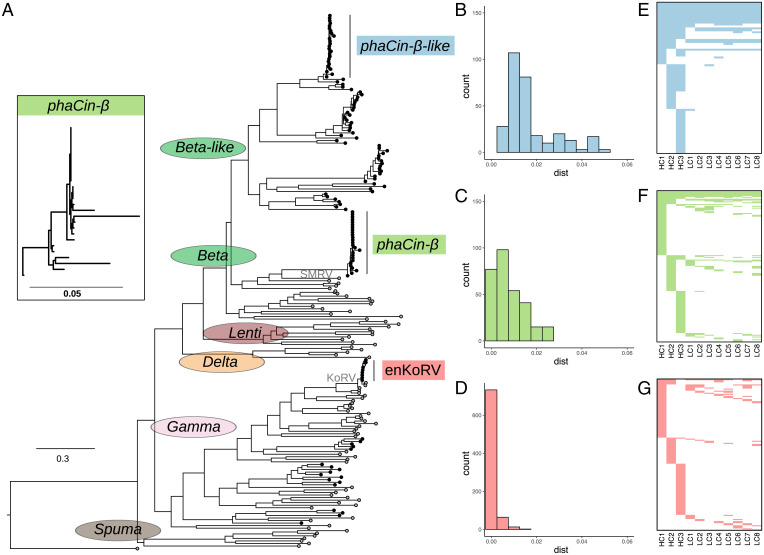
Expansion of three distinct ERV lineages in koala. (*A*) Phylogenetic tree constructed from ERVs identified in the koala genome assembly (indicated by black branch tips) and retrovirus- and ERV-reference sequences (indicated by gray branch tips), with expansions in the *phaCin-β–like*, *phaCin-β*, and KoRV lineages highlighted. The *Inset* clade shows magnified *phaCin-β* branches. (*B–D*) Histograms of genetic distances between ERV sequences in respective focal ERV expansion lineages: (*B*) the *phaCin-β–like* expansion lineage, (*C*) the *phaCin-β* expansion lineage, and (*D*) KoRV expansion. (*E–G*) Heatmaps of ERV locus presence (dark coloring) across three HC individuals (HC1–3) and eight LC individuals (LC1–8) for (*E*) the *phaCin-β–like* expansion lineage, (*F*) the *phaCin-β* expansion lineage, and (*G*) KoRV expansion. Each row in the heatmap represents an ERV locus, each column an individual, dark shading presence of the ERV in the individual. ERV loci are sorted to visualize locus-sharing between the HC individuals.

Using the koala reference genome assembly (15) and available koala whole-genome resequencing datasets, we characterized the recently expanding *phaCin-β* ERV lineage in koala, which is related to the SMRV. We present this expansion in the context of the genomic signatures of the ongoing KoRV expansion and a similar, but earlier, expansion of distantly related ERVs in koala. A total of 991 candidate ERVs were identified using the RetroTector software (scores ≥300) in the reference koala genome assembly (GCA_002099425.1: phaCin_unsw_v4.1) (15) and named using the prefix “pCi” followed by incremental numbers. To explore ERV relationships, we could align 129 high-confidence ERVs together with a catalog of reference retroviral sequences and ERVs, using previously described criteria based on conserved amino acid motifs encoded by the *gag*, *pro*, and *pol* genes, to generate a phylogenetic tree (Fig. 1*A* and Datasets S1 and S2). The resulting phylogenetic tree revealed an unexpected ERV expansion in koala that clustered together with *Betaretroviruses*, with SMRV as the most closely related reference retrovirus. Consequently, we designated these ERVs as koala *phaCin-β*. This *phaCin-β* cluster displays a wide, shallow topology, comparable to that of the KoRV clade (Fig. 1*A*), which is currently colonizing the koala germline as endogenous enKoRV. The phylogenetic analysis also revealed an expansion of *phaCin-β–like* ERVs in koala, with a branch topology suggesting expansion, but predating both the *phaCin-β* and KoRV clades (referred to as the *phaCin-β–like* expansion lineage). We investigated these three expansions further with focus on establishing the context of the koala *phaCin-β* ERVs.

**Fig. 2.**
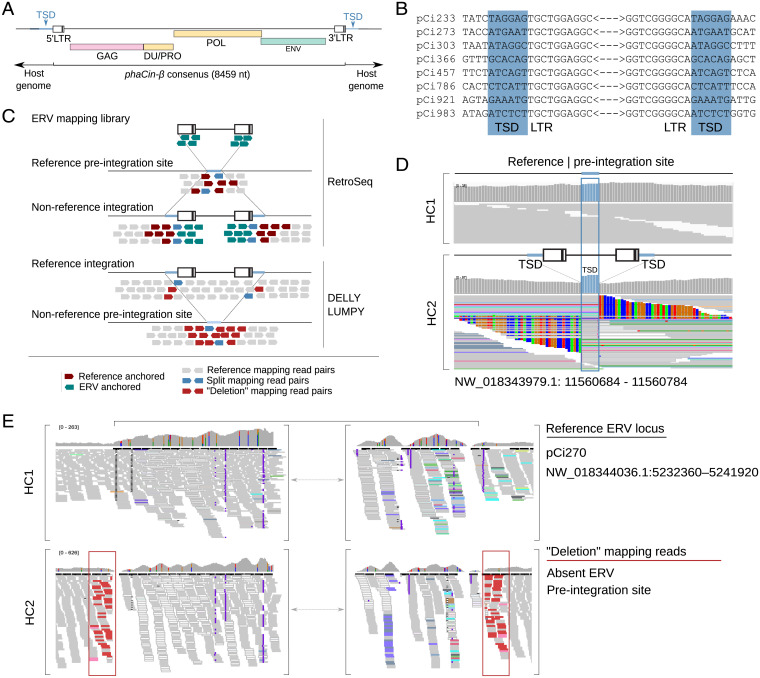
Koala *phaCin-β* ERV identification and annotation. (*A*) Schematic of the koala *phaCin-β* ERV consensus. (*B*) Sequence information for 8 *phaCin-β* ERV integration sites, identified in the koala reference genome, including 10 bp flanking either side of the abbreviated ERV, with highlighted chromosomal TSD. (*C*) Schematic overview of ERV polymorphism mapping strategy and software used. (*D*) IGV snapshot demonstrating polymorphism of a nonreference *phaCin-β* ERV integration present in HC2 but not in HC1. The *Upper* panel for each individual displays read coverage per base, while the *Lower* panel displays read information. The TSD, highlighted in blue, is characterized by heightened read coverage across these six base pairs. Soft-clipped reads on either side of the TSD (mismatching bases are color-coded by IGV) reflect reads that continue into the ERV. (*E*) IGV snapshot showing polymorphism of reference *phaCin-β* ERV locus (pCi270 NW_018344036.1:5232965–5241393). This ERV locus was predicted by RetroTector and contains a full complement of *Betaretrovirus* motifs (5LTR PBS MA CA NC DU Prot RT IN SU TM PPT 3LTR) (Dataset S1). It is present in the homozygous state in HC1. Highlight red-colored reads in HC2 predict the presence of a deletion relative to the reference genome; thus, HC2 does not contain the ERV locus (see another polymorphic locus in *SI Appendix,* Fig. S3). Note that reads mapped within the predicted deleted region are mismapped due to the high sequence similarity to *phaCin-β* ERVs and presence of multiple other *phaCin-β* integrations at other genomic locations in HC2. (IGV was downsampled 200-bp windows, 20 reads for visualization).

The phylogenetic analysis identified 26 *phaCin-β* ERVs, of which 21 were full length. Agreeing with the phylogenic annotations (Fig. 1*A*), the *phaCin-β* consensus sequence displayed a predicted full complement of *gag*, *pro*, *pol*, and *env* genes flanked by LTRs, as well as a detected dUTPase (DU), conforming to a canonical endogenous *Betaretrovirus* gene structure (16) (Fig. 2*A*). We also identified target site duplications (TSDs), features formed immediately flanking the LTRs during integration into host DNA, confirming the integration sites for the three ERV lineages (Fig. 2*B* and *SI Appendix*, Fig. S1).

The phylogenetic tree suggested deep divergence between the recently expanded *phaCin-β* ERVs and the reference SMRV sequence. Average sequence identities were consistently higher within the *phaCin-β* clade compared to the SMRV for the *gag* (97% and 46%, respectively), *pro* (92% and 53%), *pol* (95% and 56%), and *env* (80% and 56%) genes (Dataset S3). Phylogenetic comparison between *env* and *gag-propol* (Fig. 1*A*) suggests that *phaCin-β* is a recombinant that acquired a *Gammaretrovirus env* gene, similar to the related SMRV and Mason Pfizer monkey virus (*SI Appendix*, Fig. S2). Genetic distance within the koala *phaCin-β* ERV lineage (0.0 to 2.6%) was slightly greater than that in KoRV (0.0 to 1.6%) (Fig. 1 *B–D*). Genetic distances within the *phaCin-β–like* expansion lineage were greater still (0.3 to 4.9%). Divergences between the 5′- and 3′-LTRs, which are identical at the time of proviral integration, can serve as alternative estimates of relative ages for the *phaCin-β–like* expansion lineage (0 to 6.1%; average 2.5%; median 2.2%), *phaCin-β* (0 to 6.4%; average 1.4%; median 0.6%), and KoRV (0 to 0.4%; average 0.1%; median 0%).

Assuming a koala mutation rate between human (2 × 10^−9^) and the faster evolving mouse (4.5 × 10^−9^), the median LTR divergences suggest koala germline infiltration from now to between 2.4 and 5.5 million y ago for *phaCin-β*–*like*, and from now to between 0.7 and 1.5 million y ago for *phaCin-β*. These ranges predate previous estimates of KoRV colonization anytime from now to between 22,000 and 50,000 y ago (10). Accumulated inactivating mutations (stops and frameshifts) in the *pol* gene, which represents the most conserved amino acid motifs used in the phylogenetic analyses (11 in reverse transcriptase, RT, and 9 in integrase, IN) (Fig. 1*A*), correlated with the estimated relative clade ages for *phaCin-β–like* (0 to 9; average 3.8; median 4.0), *phaCin-β* (0 to 7; average 1.3; median 1.0), and KoRV (0 to 4; average 1.0; median 1.0) (*SI Appendix*, Table S1). These patterns indicate differences in colonization times of the koala germline: KoRV is the most recent, *phaCin-β* lineage is older, and the *phaCin-β-like* ERV lineage is oldest. An active *phaCin-β* retrovirus cannot be excluded based on these divergence comparisons between the *phaCin-β* ERV lineage and the KoRV/enKoRV lineage.

To characterize ERV polymorphism in koala relating to the three expanded linages, we accessed publicly available short-read whole-genome sequencing (WGS) data for three individuals with high sequencing read coverage (37 to 85×; HC1–3), and eight additional individuals with low sequencing read coverage (∼6×; LC1–8) (see *SI Appendix*, Tables S2–S4 for accession and sequencing coverage information). We generated a curated ERV mapping library (Dataset S1) from ERVs identified in the koala reference genome and representative reference sequences spanning the retrovirus phylogeny (Fig. 1*A* and Dataset S2), and used this to identify assembly and nonassembly ERV loci (Fig. 2*C*). Assembly ERVs that are not present in the resequenced individuals (i.e., their chromosomes are in the preintegration state) were identified by overlapping regions with similarity to the ERV mapping library found in the koala genome and deletions called by a combination of the DELLY (17) and LUMPY (18) softwares (Fig. 2*D*). Nonassembly ERVs were identified in the resequenced individuals by the RetroSeq. (19) software using the ERV mapping library and short reads as input (Fig. 2*E*).

Focusing on the ERV lineage expansions above, we identified 392 ERV loci in the 11 koala individuals, of which 48 loci were present in the reference assembly and 344 were nonreference loci (Table 1). Most loci were identified either as *phaCin-β* (169 loci) or as KoRV (145 loci), compared to the *phaCin-β–like* expansion lineage (78 loci). For all three lineages, the number of loci varied across the HC individuals (33 to 58 *phaCin-β–like*; 57 to 73 *phaCin-β*; 30 to 56 KoRV loci) (*SI Appendix*, Table S2), indicating substantial polymorphism (Fig. 1 *E–G*). As expected from the inferred relative ERV lineage ages (Fig. 1 *B–D*), we found a greater degree of locus sharing among the koalas for the *phaCin-β–like* lineage, where 56% of loci were observed in more than one individual, compared to 34% of *phaCin-β* loci and 26% of KoRV loci (Fig. 1 *E–G*). An earlier age for the *phaCin-β–like* expansion lineage allows more time for shared ERV loci to accumulate in the population. We note that these observations underestimate locus sharing and overall loci counts due to lower detection power in the LC compared to HC individuals, which likely affect the three expansions equally.

**Table 1.** Numbers of identified loci for the three focal ERV expansion lineages in koala

ERV lineage	Identified loci	Assembly ERV loci (frequency range/mean)	Nonassembly ERV loci (frequency range/mean)
*phaCin-β-like*	78	15 (10–11 / 10.8)	63 (1–9 / 1.8)
*phaCin-β*	169	23 (1–11 / 5.7)	146 (1–6 / 1.6)
enKoRV	145	10 (1–9 / 2.6)	135 (1–6 / 1.4)

**Fig. 3.**
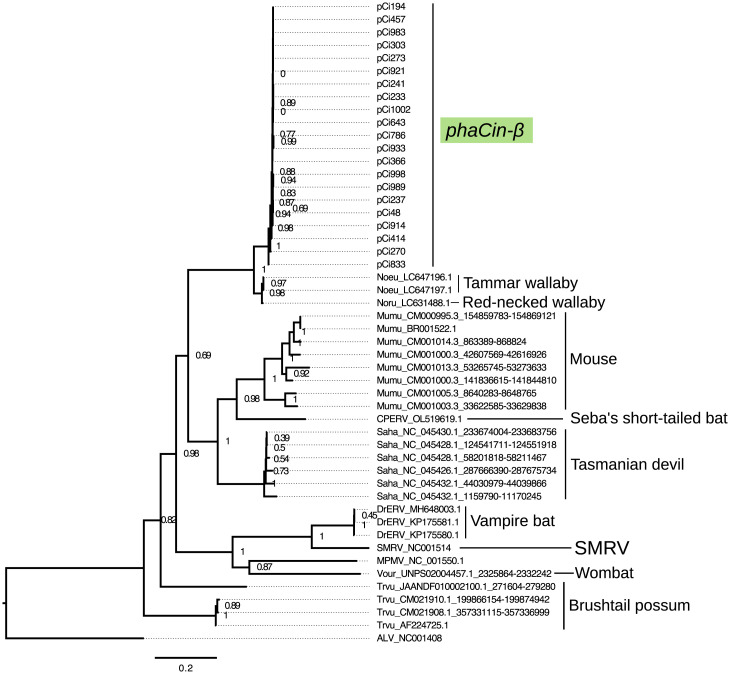
*Pol* phylogenetic tree of *phaCin-β,* SMRV, and related sequences from similarity searches and the literature, rooted on avian leukemia virus (ALV). The *phaCin-β* ERVs *pol* genes form a distinct clade next to wallaby sequences, showing deep divergence to the SMRV and related ERVs from marsupial, bat, and murine species (CPERV, *Carollia perspicillata*; DrERV, *Desmodus rotundus*; Mumu, *Mus musculus*; Noeu, *Notamacropus eugenii*; Noru, *Notamacropus rufogriseus*; Sahar, *Sarcophilus harrisii*; Trvu, *Trichosurus vulpecula*; Vour, *Vombatus ursinus*).

To explore a potential transmission history, we searched for *phaCin-β* ERVs in other marsupial genomes, with positive results in Tammar wallaby (*Notamacropus eugenii*), red-necked wallaby (*Notamacropus rufogriseus*), brushtail possum (*Trichosurus vulpecula*), Tasmanian devil (*Sarcophilus harrisii*), and common (bare-nosed) wombat (*Vombatus ursinus*). *phaCin-β* similarity was also found in the house mouse (*Mus musculus*) genome, including matches to the ERV implicated in the black coat (nonagouti) phenotype in the East Asian mouse genome (20). Nucleotide BLAST (21) of the koala *phaCin-β* sequence (pCi643) (Dataset S1) returned highly confident hits (>84% sequence identity; up to 95% query coverage) to wallaby retrotransposon sequences isolated from Japanese zoo individuals (22). A more detailed *pol* phylogeny, including these and additional SMRV-like sequences from the literature (23, 24), breaks up the deep divergence to SMRV, confirming that *phaCin-β* is a distinct clade (Fig. 3). A more closely related *phaCin-β* retrovirus, the existence of which is implied by the expansion in koala and presence in wallaby (Fig. 3), remains to be discovered. Given these results, it is conceivable that multiple host species, and marsupials in particular, share evolutionary history with *phaCin-β* retroviruses in the Australasian region.

## Discussion

Here, we present conclusive evidence for recent *phaCin-β* retrovirus activity in koala by utilizing the genomic ERV record and identifying numerous ERV loci with high sequence similarity and shallow phylogenetic branching. Although the study utilized limited samples with modest sequencing coverage, the degree of locus sharing is evidence for ERVs, as opposed to naturally occurring somatic integrations during retroviral replication. Additional koala datasets will improve detection power and identify further shared ERV loci, as well as allowing for estimation of their frequencies in the host population.

The expansion of *phaCin-β* ERVs in koala is comparable to that of the KoRVs, with a similarly shallow phylogenetic topology and intraclade genetic distances as low as 0%. This observation reflects very recent, possibly even ongoing, *phaCin-β* retrovirus activity in koalas. All individuals in this study had more *phaCin-β* loci than KoRV, and the maximum intraclade genetic distances were greater in the *phaCin-β* lineage, which suggests that the start of *phaCin-β* activity predates the KoRV invasion of the koala germline that occurred within the last 50,000 y (10).

The impact of *phaCin-β* activity on koala biology remains unclear. Presence of *phaCin-β* ERVs in other host species (3) and marsupial genomes described here implies historical activity of *phaCin-β* retrovirus replication in Australia, and could reflect contemporary infection. Isolating and characterizing a koala *phaCin-β* retrovirus will be an important step in understanding infection dynamics and its evolutionary history in koala and the wider marsupial phylogeny. There is a chance, however, that such retroviruses may be recently extinct, as documentation of *phaCin-β* retrovirus infection is notably absent, despite intensive koala surveillance.

Functional consequences of *phaCin-β* integrations into the koala genome also remain unknown, but a diminished strength of selection in small host populations could allow slightly deleterious ERVs to persist, or even increase in frequency due to genetic drift or founder effects from koala translocations. Endogenous enKoRV can mediate a predisposition to tumors (25), and likewise, the potential negative health impacts of *phaCin-β* ERVs may prove especially important for the conservation management of the koala. Population genomic studies will be important to understand the geographic distribution of *phaCin-β* ERVs in the koala population, how this relates to KoRV, and whether correlation exists between the respective viruses.

This study demonstrates the strength of applying broad genomics-based ERV analyses for valuable insights into past retrovirus–host dynamics. Our results strongly suggest the occurrence of at least two recent, possibly ongoing, retroviral invasions of the koala population and predicts novel *phaCin-β* retroviruses in the Australasian fauna. Additional insights into historic retrovirus–host associations can be obtained from extending the approach in this study to WGS datasets of other natural populations.

In conclusion, we present considerable evidence for recent *phaCin-β* retrovirus activity in koala, resulting in an expansion of *phaCin-β* ERVs in koala genomes. These findings generate a strong incentive for a new area of research into *phaCin-β* retroviruses, and we anticipate future ERV-guided discovery of novel viruses along these lines in a variety of host species.

## Materials and Methods

### Koala Assembly ERVs.

ERVs were identified in the koala reference genome assembly (GCF_002099425.1_phaCin_unsw_v4.1) using the RetroTector software (26), as previously described (3, 27, 28). This approach identified 991 ERVs (RetroTector scores ≥300) labeled as “pCi” and sequential numbering, which were further pruned by phylogenetic analysis using maximum likelihood in FastTree2 (29), specifying the GTR model of nucleotide sequence evolution, based on sequence alignment of conserved amino acid motifs in the *gag* (two motifs in matrix; two in capsid; two in nucleocapsid), *pro* (two from protease), and *pol* (11 in RT and 9 in IN) genes, as previously described (3, 27, 28). The final alignment covered 1,502 nt. The resulting phylogenetic tree included 129 koala ERV sequences, as well as previously identified KoRV sequences and other reference retroviruses spanning the retroviral phylogeny (Datasets S1 and S2). Three focal ERV lineage expansions *(phaCin-β–like*, *phaCin-β*, and enKoRV) showed varying intraclade divergences calculated from pairwise identity using complete ERV sequence comparisons generated from multiple sequence alignments in MUSCLE (30).

### Koala Samples.

Whole-genome resequencing data from koalas was accessed from the Sequence Read Archive (SRA; https://www.ncbi.nlm.nih.gov/sra) for exploring ERV polymorphism. The data included three individuals sequenced at high coverage (HC1–3; note that HC1 is the koala reference genome individual) as well as eight individuals with lower sequencing coverage (LC1–8) (accession information listed in *SI Appendix*, Table S3; all analyzed samples derive from northern koala populations). Sequencing reads were mapped to the koala reference using BWA-MEM (31), pooled per individual with SAMtools 1.12 (32), and duplicate reads marked by Picard 2.23.4 (broadinstitute.github.io/picard/). Coverage per individual pool was estimated with SAMtools depth (*SI Appendix*, Table S4). Integrative genomics viewer (IGV) (33) was used to inspect and visualize sequencing read alignments.

### Assembly ERV Integration Mapping.

Polymorphism among reference ERVs was identified by overlapping ERV candidates found in the reference genome with called deletions across the resequenced individuals. The reference ERV candidate dataset was the combination the RetroTector called ERV (pCi) dataset, regions identified by BLAT (34) with similarity to full length ERVs in the three focal expansions (nonoverlapping with RetroTector called loci), and regions identified by BLAT with similarity to the LTRs of ERVs in the three focal expansions (nonoverlapping with the two previous datasets). Deletions were called in the resequenced individuals with DELLY (17) and LUMPY (18). Deletion calls overlapping reference ERV candidates were then identified in R (35) (GitHub: https://github.com/PatricJernLab/Koala_ERVs). Putative loci were defined using stringent filters, but relaxed for ERV typing of individuals.

### Nonassembly ERV Integration Mapping.

Nonreference ERV loci were detected by RetroSeq. (19) using a custom ERV reference library (with the “-eref” flag). This library was formed from sequences included in the phylogenic analysis (above), with koala ERVs restricted to those that had both LTRs (100 pCi ERV sequences) and all hard-masked for simple repeats and low-complexity regions by RepeatMasker (36) to limit false-positive mapping. RetroSeq call applied soft-clips and five reads for the HC samples and with soft-clips and two reads for the LC samples. ERV loci with stringent RetroSeq filters (FL8; CLIP3 ≥ 2; CLIP5 ≥ 2; cov/2 <= minGQ <= 3*cov) were called in R (https://github.com/PatricJernLab/Koala_ERVs). Loci that overlapped with RetroTector-identified ERVs, or RepeatMasker (36)-detected simple repeats or regions of low complexity, were removed from analysis. RetroSeq filters were relaxed (FL3; GC1) to count common ERV integrations across individuals.

Scrutinizing soft-clipped reads in the low-sequencing coverage individuals around the defined ERV loci allowed for improvement of calling therein. Soft-clipped reads within 300-bp upstream and 300-bp downstream of ERV loci were pulled from BAM files using the samextractclip java software in jvarkit (37) (-m 10). Soft-clipped sequences with similarity to ERV LTR sequences were identified by BLAT (-tileSize = 8, -minIdentity = 80, -minScore = 10), and all results were summarized in R in order to adjust counts. This annotation approach slightly improved our ability to detect ERV loci in the low-sequence coverage samples.

Sequences related to the SMRV reference and koala *phaCin-β* ERVs were mined using BLAT similarity searches (blat -minIdentity = 80 -minScore = 300 -noHead -oneOff = 1) in mammal genomes downloaded from public servers, including marsupials: Tammar wallaby (Meug 1.1, GCA_000004035.1), brushtail possum (mTriVul1, GCA_011100635.1), Tasmanian devil (mSarHar1.11, GCF_902635505.1), common (bare-nosed) wombat (UNPS02, GCA_900497805), and the house mouse (GRCm39, GCA_000001635.9). One *phaCin-β* sequence (pCi643) (Dataset S1) was also used in nucleotide BLAST (21) (nr/nt BLASTN) to search for *phaCin-β* similarity in online databases. Phylogenetic relationships of identified sequences were confirmed using MUSCLE (30) and FastTree2 (29).

## Supplementary Material

Supplementary File

Supplementary File

Supplementary File

Supplementary File

## Data Availability

Code and supporting files are available at GitHub: (https://github.com/PatricJernLab/Koala_ERVs). All other study data are included in the main text and supporting information.
