# Supplementary material for: Expansion of a retrovirus lineage in the koala genome
Source: Proc Natl Acad Sci U S A. 2022 Jun 13;119(25):e2201844119. doi: 10.1073/pnas.2201844119 (PMC9231498; doi:10.1073/pnas.2201844119)
Supplement: Supplementary File [file pnas.2201844119.sapp.pdf]

PNAS
www.pnas.org

Supplementary Information for

Expansion of a retrovirus lineage in the koala genome

Mette Lillie[a,1], Jason Hill[a], Mats E. Pettersson[a], and Patric Jern[a,1]

[a] *Science for Life Laboratory, Department of Medical Biochemistry and Microbiology, Uppsala University, SE-751 23, Uppsala, Sweden*

[1] Correspondence:

Mette Lillie
Email: mette.lillie@imbim.uu.se

Patric Jern
Email: patric.jern@imbim.uu.se

This PDF file includes:

Figures S1 to S3
Tables S1 to S4

Other supplementary materials for this manuscript include the following:

Datasets S1 to S3

phaCin-β -like

pCi34	TCTC**CCCACA**TGTTGAGGGC<--->GTGGGTGACA**CCCACA**CCCT
pCi90	CATG**CTTCTC**TGTTGAGGGC<--->GCGGGCAACA**CTTCTC**TGTA
pCi147	GATT**AGGGAA**TGTTTAGGGC<--->GCGGGCAACA**AGGGAA**TCCT
pCi170	CTAT**CTGGAG**CGTTGAGGGC<--->GCAGGCAACA**CTGGAG**TATT
pCi301	ATTG**GTCAAG**AGTTGAGGGC<--->GCGGGCAACA**GTCAAG**AGAA
pCi319	CATT**ATGTG**CTGTTGAGGGC<--->GCGGGCAACA**ATGTG**ATTTC
pCi320	AGTC**ACTTAA**AGTTGAGGGC<--->GCGGGCAATA**ACTTAA**CCCT
pCi321	GAAA**GCTTTT**TGTTGAGG-C<--->GCGGGCAACA**GCTTTT**CTCA
pCi387	TCAG**GTATCT**TGTTGAGGGC<--->GCGGGCAACA**GTATCT**TAAA
pCi421	TTGT**CTTTCA**TGTTGAGGGC<--->GTGGGCAACA**CTTTCA**CTTT
pCi449	GGAG**GAGGAA**TGTTGAGGGC<--->GCGGGCAACA**GAGGAA**GAGC
pCi471	TTA**TGGAAAG**TGTTGAGGGC<--->GCGGGCAACA**TGGAAAG**TTA
pCi491	AGAG**CTTAAC**TGTTGAGGGC<--->GCGGGCAACA**CTTAAC**CCAA
pCi494	GAAA**GGGAGA**TGTTCAGGGC<--->GCAGGCGACA**GGGAGA**GGTA
pCi514	ATTC**CATGGA**TGCTGAGGGC<--->GCAGGCAACA**CATGGA**TGAT
pCi521	ACTT**CTGACA**TGTTGAGGGC<--->GCGGGCGACA**CTGACA**CTGA
pCi571	TCCT**GTTTGC**TGTTGAGGGC<--->GCGGGCAACA**GTTTGC**CATT
pCi577	GAAG**GGTTTT**TGTTGAGGGC<--->GTGGACAACA**GGTTTT**AGGC
pCi667	ATGCT**GGATG**TGTTGAGGGC<--->GCGGGCAACA**GGATG**ACCAG
pCi753	ATTC**CAACCC**TGTTGAGGGC<--->GCGGGCAACA**CAACCC**AACA
pCi760	ATTT**GGAAAG**TGTTGAGGGC<--->GCGGGCAACA**GGAAAG**AGAG
pCi797	TCAC**CATCAT**TGTTGAGGGC<--->GCAGGCAACA**CATCAT**TATG
pCi844	CTAC**CTCTCC**TGTTGAGGGC<--->GCGGGCAACA**CTCTCC**CTTT

phaCin-β

pCi47	AAAA**GTGTCC**TGCTGGAGGC<--->GGTCGGGGCA**GTGTCC**ATGA
pCi48	TGAT**ATAATT**TGTTGGAAGC<--->GGTCGGGGCA**ATAATT**ATGG
pCi71	ACTC**GATAAA**TGTTGGAGGC<--->GGTCGGGGCA**GATAAA**TGCT
pCi194	TGCC**TTCCTG**TGTTGGAGGC<--->GGTCGGGGCA**TTCCTG**AGTT
pCi233	TATC**TAGGAG**TGCTGGAGGC<--->GGTCGGGGCA**TAGGAG**AAAC
pCi237	GAAG**GGGAGG**TGTTGGAGGC<--->GGTCGGGGCA**GGGAGG**ATAC
pCi241	GCTC**AAAAAG**TGTTGGAGGC<--->GGTCGGGGCA**AAAAAG**CCTT
pCi270	AATT**CTTCTT**TGTTGGAGGC<--->GGTCGGGGCA**CTTCTT**GATA
pCi273	TACC**ATGAAT**TGCTGGAGGC<--->GGTCGGGGCA**ATGAAT**GCAT
pCi303	TAAT**ATAGGC**TGCTGGAGGC<--->GGTCGGGGCA**ATAGGC**CTTT
pCi366	GTTT**GCACAG**TGCTGGAGGC<--->GGTCGGGGCA**GCACAG**AGCT
pCi414	CATA**CATACA**TGTTGGAGGC<--->GGTCGGGGCA**CATACA**GACT
pCi419	TATT**GTATAT**TGTTGGAGGC<--->GGTCGGGGCA**GTATAT**AGTC
pCi457	TTCT**ATCAGT**TGCTGGAGGC<--->GGTCGGGGCA**ATCAGT**CTCA
pCi643	TATA**GATAGA**TGTTGGAAGC<--->GGTCGGGGCA**GATAGA**TAGA
pCi786	CACT**CTCATT**TGCTGGAGGC<--->GGTCGGGGCA**CTCATT**TCCA
pCi833	CTTG**CTTTAC**TGTTGGAGGC<--->GGTCGGGGCA**CTTTAC**AAGT
pCi880	GGCA**GATGTA**TGCTGGAGGC<--->GGTCGGGGCA**GATGTA**AATA
pCi914	CTTA**ATGGTG**TGTTGGAAGC<--->GGTCGGGGCA**ATGGTG**TGCT
pCi921	AGTA**GAAATG**TGCTGGAGGC<--->GGTCGGGGCA**GAAATG**ATTG
pCi933	AATG**TTAAAG**TGTTGGAGGC<--->GGTCGGGGCA**TTAAAG**TAGC
pCi983	ATAG**ATCTCT**TGCTGGAGGC<--->GGTCGGGGCA**ATCTCT**GGTG
pCi989	CATG**AAACAT**TGCTGGAGGC<--->GGTCGGGGCA**AAACAT**AAAC
pCi998	CCAT**AATAGC**TGTTGGAAGC<--->GGTCGGGGCA**AATAGC**CATT
pCi1002	AGTG**GGCCAA**TGCTGGAGGC<--->GGTCGGGGCA**GGCCAA**TGTT

enKoRV

pCi17	AGGAC**AATAT**TGAAGGAGGC<--->GGGTCTTTCA**AATAT**GAGTT
pCi138	ATAAT**GTGGG**TGAAGGAGGC<--->GGGTCTTTCA**GTGGG**GAGGG
pCi140	AAACT**GGCTA**TGAAGGAGGC<--->GGGTCTTTCA**GCTAC**AAGAT
pCi365	CAGCAG**ACAT**TGAAGGAGGC<--->AGGTCTTTCA**ACAT**GAAGGA
pCi525	TAAGAC**AAGG**TGAAGGAGGC<--->GGGTCTTTCA**AAGG**AAAAAT
pCi617	CGAGAG**CAAG**TGAAGGAGGC<--->GGGTCTTTCA**CAAG**GAATAG
pCi954	TAGCTC**ATTT**TGAAGGAGGC<--->GGGTCTTTCA**ATTT**CAAGAC
pCi988	TTGGCC**ATGG**TGAAGGAGGC<--->GGGTCTTTCA**ATGG**GTTTCT

Fig. S1. ERV integration target site duplications (TSDs) in the koala assembly. Manually curated ERV integration sites in the koala assembly for the phylogenetically determined *phaCin-β-like* expansion lineage, *phaCin-β* and enKoRV (Fig. 1). Integration target site duplications (TSDs) are underlined in bold font immediately flanking to the abbreviated ERV 5'- and 3'-LTR (long terminal repeat) ends indicated in blue.

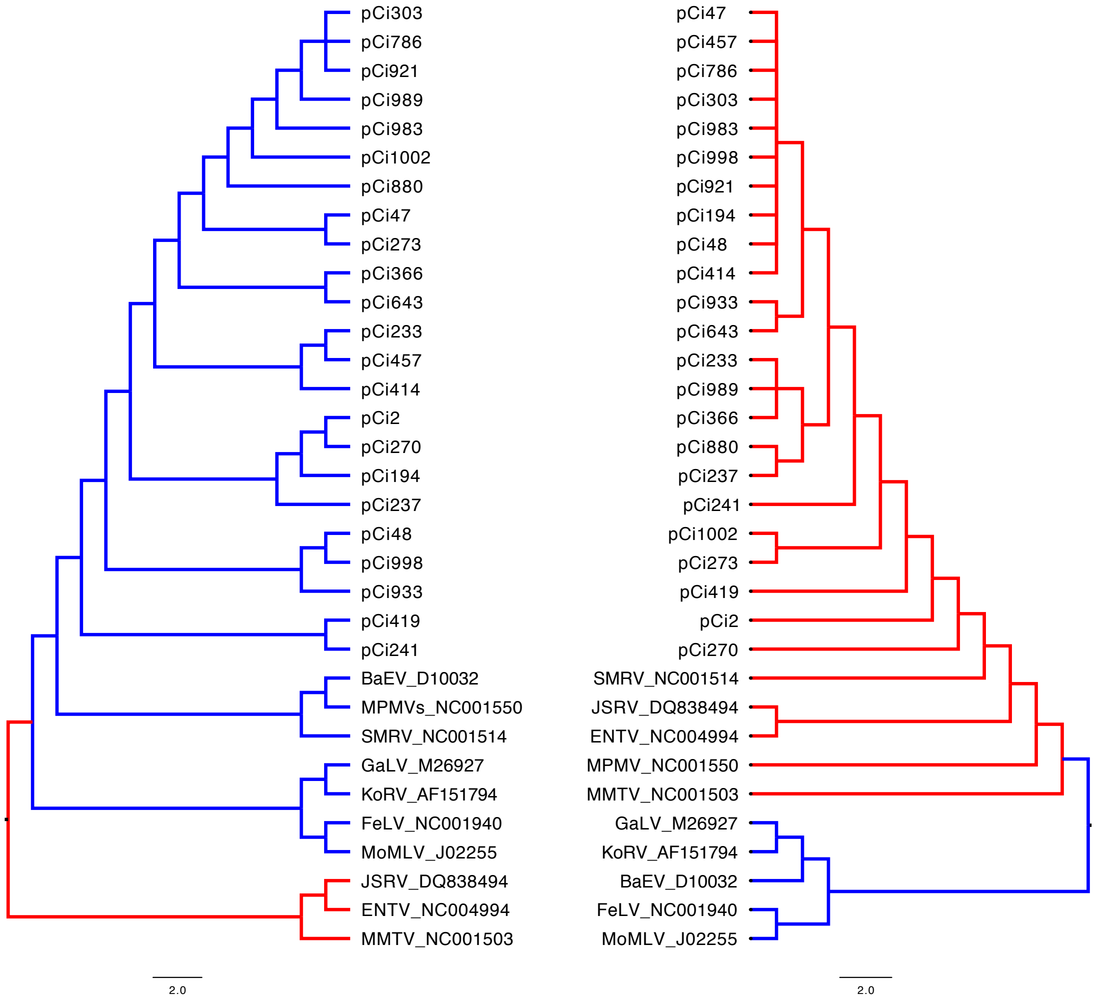

Fig. S2. The *phaCin-β* ERVs *env* genes group with *Gammaretroviruses*. The left cladogram (*env*) indicates that *phaCin-β* groups together with *Gammaretroviruses* (blue branches) suggesting recombinant origin and acquisition of *Gammaretroviral env* similarly to MPMV and SMRV, which like the *phaCin-β* ERVs group as *Betaretroviruses* (red branches) in the *gag-pro-pol* based cladogram (right panel, truncated from Fig. 1*A,* Dataset S2).

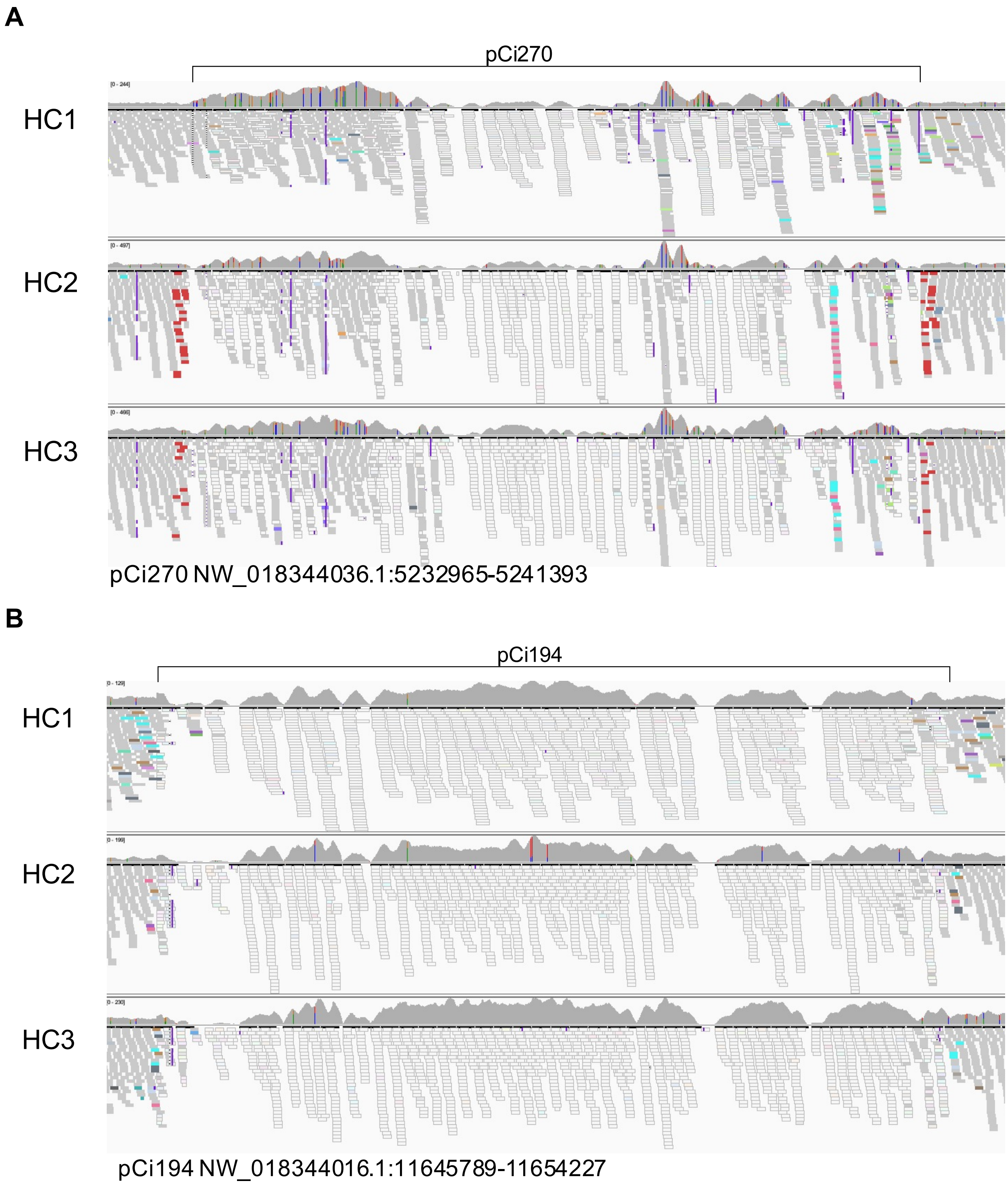

Fig. S3. Examples of reference ERV polymorphism. IGV screen grabs for *phaCin-β* ERV mapping loci in koala. (*A*) Homozygous locus in the reference assembly (HC1), missing in HC2 as indicated by red-flagged reads (Fig. 2*C*), and heterozygote in HC3 indicated by present red-flagged reads albeit fewer than in HC2. (*B*) Example of a fixed homozygous pCi194 associated locus in HC1, HC2 and HC3 as indicated by populated grey and white mapping reads across the ERV, and absence of any red-flagged reads at boundaries.

Table S1. Inactivating mutations in koala ERV expansion clades

ERV id[1]	Clade	*gag*		*pro*		*pol*		*env*		LTR
		Stops	Shifts	Stops	Shifts	Stops	Shifts	Stops	Shifts	(% div)
pCi421	*phaCin-β-like*	3	5	0	1	1	3	0	0	2,41
pCi760	*phaCin-β-like*	0	2	0	1	0	0	0	0	1,30
pCi170	*phaCin-β-like*	0	1	0	0	0	2	0	0	0,95
pCi494	*phaCin-β-like*	0	3	0	2	0	5	nd[2]	nd	2,82
pCi321	*phaCin-β-like*	0	2	0	3	1	4	nd	nd	2,17
pCi521	*phaCin-β-like*	1	3	0	2	0	1	nd	nd	0,47
pCi387	*phaCin-β-like*	0	3	0	1	2	7	nd	nd	1,96
pCi449	*phaCin-β-like*	0	1	0	1	2	3	nd	nd	2,40
pCi147	*phaCin-β-like*	3	1	0	3	0	3	nd	nd	4,52
pCi471	*phaCin-β-like*	0	2	0	0	0	1	nd	nd	2,28
pCi844	*phaCin-β-like*	0	0	0	1	1	3	0	0	5,07
pCi34	*phaCin-β-like*	0	1	0	2	2	2	nd	nd	1,96
pCi319	*phaCin-β-like*	3	4	0	1	1	5	nd	nd	3,90
pCi320	*phaCin-β-like*	0	1	0	1	0	3	nd	nd	2,84
pCi301	*phaCin-β-like*	0	3	0	0	3	4	nd	nd	4,15
pCi491	*phaCin-β-like*	0	3	0	3	0	6	0	0	6,12
pCi467	*phaCin-β-like*	0	2	0	1	2	4	nd	nd	nd
pCi571	*phaCin-β-like*	3	1	1	2	0	2	nd	nd	1,08
pCi753	*phaCin-β-like*	0	0	0	1	0	1	nd	nd	1,52
pCi577	*phaCin-β-like*	0	4	0	2	0	1	nd	nd	3,34
pCi667	*phaCin-β-like*	0	1	0	0	2	2	nd	nd	1,95
pCi797	*phaCin-β-like*	0	3	0	1	0	3	nd	nd	0,65
pCi90	*phaCin-β-like*	1	4	0	1	1	2	1	0	1,13
pCi514	*phaCin-β-like*	1	1	0	3	1	6	nd	nd	1,96
pCi15	*phaCin-β-like*	1	4	0	3	1	2	nd	nd	nd
pCi47	*phaCin-β*	0	0	0	0	0	0	2	2	0,00
pCi457	*phaCin-β*	0	0	0	0	0	0	0	2	0,00
pCi786	*phaCin-β*	0	0	0	0	0	0	1	3	0,00
pCi303	*phaCin-β*	0	1	1	0	0	0	1	3	0,31
pCi983	*phaCin-β*	0	0	0	0	0	0	1	3	0,00
pCi998	*phaCin-β*	0	4	1	1	0	1	3	5	6,37
pCi921	*phaCin-β*	0	0	0	0	0	0	1	3	0,00
pCi194	*phaCin-β*	0	2	1	0	0	0	0	4	1,55
pCi48	*phaCin-β*	0	9	0	3	0	1	3	6	3,47
pCi414	*phaCin-β*	0	3	1	1	0	0	0	4	1,86
pCi233	*phaCin-β*	0	0	0	1	0	1	0	1	0,00
pCi989	*phaCin-β*	0	1	0	1	0	2	1	5	0,00
pCi366	*phaCin-β*	0	1	1	0	0	1	2	5	0,00
pCi880	*phaCin-β*	0	0	1	1	0	1	2	2	0,00
pCi914	*phaCin-β*	1	9	1	2	0	5	nd	nd	4,21
pCi71	*phaCin-β*	1	7	1	4	0	2	1	0	2,48
pCi643	*phaCin-β*	0	3	1	1	0	1	0	6	3,19
pCi933	*phaCin-β*	3	9	0	3	0	1	1	2	1,55
pCi237	*phaCin-β*	1	5	1	2	0	4	0	4	2,81
pCi241	*phaCin-β*	2	7	1	2	0	1	1	6	0,58
pCi273	*phaCin-β*	1	4	0	0	0	0	2	4	0,00
pCi1002	*phaCin-β*	0	2	1	0	0	1	1	3	0,00
pCi419	*phaCin-β*	0	2	0	4	0	2	2	7	0,60
pCi2	*phaCin-β*	nd	nd	0	1	0	3	4	8	nd
pCi833	*phaCin-β*	0	1	1	0	0	1	nd	nd	3,64
pCi270	*phaCin-β*	0	3	1	3	0	7	0	6	1,79
pCi140	*enKoRV*	0	2	0	0	0	0	0	1	0,00
pCi525	*enKoRV*	0	2	0	0	0	1	0	3	0,40
pCi617	*enKoRV*	0	1	0	0	0	0	0	2	0,00
pCi365	*enKoRV*	0	0	0	0	1	3	nd	nd	nd
pCi138	*enKoRV*	0	1	0	0	0	1	0	1	0,00
pCi988	*enKoRV*	0	1	0	0	0	0	0	1	0,00
pCi17	*enKoRV*	0	0	0	0	0	1	0	0	0,20

1. ERV id in phylogeny order (Fig. 1*A*, Dataset S2)
2. nd: not determined by the RetroTector software

Table S2. Number of ERVs from three lineages identified in individual koalas

ID	Number of *phaCin-β-like* loci	Number of *phaCin-β* loci	Number of enKoRV loci
HC1	32	72	56
HC2	47	55	30
HC3	60	63	50
LC1	17	23	12
LC2	16	23	9
LC3	17	26	5
LC4	18	19	8
LC5	18	13	11
LC6	18	22	8
LC7	16	15	12
LC8	16	28	9

Table S3. SRA accession information for koala whole genome sequencing data

SRA Accession No.	Sample	Contd	
		SRA Accession No.	Sample
ERR1881585	Birke	ERR2575702	Jaffa
ERR1881586	Birke	ERR2575703	Jaffa
ERR1881587	Birke	ERR2575704	Jaffa
ERR1881588	Birke	ERR2575705	Utopia
ERR1881589	Birke	ERR2575706	Utopia
ERR1881590	Birke	ERR2575707	Utopia
ERR1881591	Birke	ERR2575708	Utopia
ERR2541467	Mintie	ERR2575709	Bertha
ERR2541468	Meander	ERR2575710	Bertha
ERR2541469	Indigo	ERR2575711	Bertha
ERR2541470	Jaffa	ERR2575712	Bertha
ERR2541471	Utopia	ERR2575713	Amity
ERR2541472	Bertha	ERR2575714	Amity
ERR2541473	Amity	ERR2575715	Amity
ERR2541474	Guppy	ERR2575716	Amity
ERR2575689	Mintie	ERR2575717	Guppy
ERR2575690	Mintie	ERR2575718	Guppy
ERR2575691	Mintie	ERR2575719	Guppy
ERR2575692	Mintie	ERR2575720	Guppy
ERR2575693	Meander	ERR3485163	Bilbo
ERR2575694	Meander	ERR409789	Pacific Chocolate
ERR2575695	Meander	ERR409790	Pacific Chocolate
ERR2575696	Meander	ERR409791	Pacific Chocolate
ERR2575697	Indigo	ERR409792	Pacific Chocolate
ERR2575698	Indigo	ERR409793	Pacific Chocolate
ERR2575699	Indigo	ERR409794	Pacific Chocolate
ERR2575700	Indigo	ERR409795	Pacific Chocolate
ERR2575701	Jaffa		

Table S4. Mapped sequence coverage for koala samples

ID	Individual	Genome x coverage	Associated project
HC1	Bilbo	37.2429	Genome project – reference genome
HC2	Birke	71.2481	Genome project
HC3	Pacific Chocolate	85.9601	Genome project
LC1	Amity	6.14109	Immunogenetics
LC2	Bertha	5.65035	Immunogenetics
LC3	Guppy	5.71537	Immunogenetics
LC4	Indigo	6.15041	Immunogenetics
LC5	Jaffa	5.99881	Immunogenetics
LC6	Meander	6.5338	Immunogenetics
LC7	Mintie	5.68336	Immunogenetics
LC8	Utopia	5.90399	Immunogenetics

Dataset S1 (separate file). ERV mapping library in FASTA file format.

Dataset S2 (separate file). Phylogenetic tree in nexus file format.

Dataset S3 (separate file). SMRV and *phaCin-β* percent identity matrices in Excel format.
